# Sorafenib Repurposing for Ophthalmic Delivery by Lipid Nanoparticles: A Preliminary Study

**DOI:** 10.3390/pharmaceutics13111956

**Published:** 2021-11-18

**Authors:** Angela Bonaccorso, Veronica Pepe, Cristina Zappulla, Cinzia Cimino, Angelo Pricoco, Giovanni Puglisi, Francesco Giuliano, Rosario Pignatello, Claudia Carbone

**Affiliations:** 1 Laboratory of Drug Delivery Technology, Department of Drug and Health Sciences, University of Catania, Viale A. Doria 6, 95125 Catania, Italy; angela.bonaccorso@unict.it (A.B.); cinzia.cimino@phd.unict.it (C.C.); puglisig@unict.it (G.P.); rosario.pignatello@unict.it (R.P.); 2 Research, Preclinical Development and Patents, SIFI S.p.A., Lavinaio-Aci S. Antonio, 95025 Catania, Italy; veronica.pepe@sifigroup.com (V.P.); cristina.zappulla@sifigroup.com (C.Z.); angelo.pricoco@sifigroup.com (A.P.); francesco.giuliano@sifigroup.com (F.G.); 3 NANO-i, Research Centre for Ocular Nanotechnology, University of Catania, Viale A. Doria 6, 95125 Catania, Italy

**Keywords:** nanomedicine, drug repurposing, Softisan 100, uveal melanoma, mucoadhesion, eye irritation test, ocular delivery

## Abstract

Uveal melanoma is the second most common melanoma and the most common intraocular malignant tumour of the eye. Among various treatments currently studied, Sorafenib was also proposed as a promising drug, often administered with other compounds in order to avoid resistance mechanisms. Despite its promising cellular activities, the use of Sorafenib by oral administration is limited by its severe side effects and the difficulty to reach the target. The encapsulation into drug delivery systems represents an interesting strategy to overcome these limits. In this study, different lipid nanoparticulate formulations were prepared and compared in order to select the most suitable for the encapsulation of Sorafenib. In particular, two solid lipids (Softisan or Suppocire) at different concentrations were used to produce solid lipid nanoparticles, demonstrating that higher amounts were able to achieve smaller particle sizes, higher homogeneity, and longer physical stability. The selected formulations, which demonstrated to be biocompatible on Statens Seruminstitut Rabbit Cornea cells, were modified to improve their mucoadhesion, evaluating the effect of two monovalent cationic lipids with two lipophilic chains. Sorafenib encapsulation allowed obtaining a sustained and prolonged drug release, thus confirming the potential use of the developed strategy to topically administer Sorafenib in the treatment of uveal melanoma.

## 1. Introduction

Among the intraocular malignant tumours affecting the inner eye, the most common in adults is uveal melanoma (UM) [1]. UM represents the second most common melanoma after the cutaneous one—with a lifetime risk of 1 in 2500 and an incidence of 6 per million per year—but the incidence and biological implications are different [2]. An important risk factor is the presence of ocular lesions, while sunlight seems not to be involved in the occurring of this condition, as it is prevalent in people living in northern Europe than in the ones living in the South. Moreover, the pigmentation could have a protective function since a risk factor is having a lightly coloured iris [3].

Characteristic of this melanoma is the great ability to metastasise (since the occurrence of the primary tumour, metastasis appear in 5 years for 25% of patients and in 10 years for 34%), and the most affected organ is the liver (90%), with lungs and soft tissues following [4]. Mortality in the metastatic UM is about 50% in one year and it is greatly influenced by the progression of liver involvement [5].

Based on the experience with cutaneous melanomas, various systemic treatments have been investigated also for uveal one and clinical trials demonstrated a modest efficacy [6].

Combinations of new drugs are currently studied to verify their suitability in the treatment of UM; it is worth mentioning bortezomib in combination with celecoxib, prednisone, temozolomide, dacarbazine, anti-angiogenic agents such as bevacizumab, sunitinib, cetuximab, panitumumab, erlotinib, transtuzumab, or temsirolimus, MEK inhibitors or ipilimumab [3].

In addition to the mentioned drugs analysed for UM, also Sorafenib (SRF) was proposed, generally in combined treatment with paclitaxel, doxorubicin, and siRNA, in order to overcome resistance mechanisms [1,2,3,7,8]. SRF is a small hydrophobic molecular inhibitor of several tyrosine protein kinases, which rapidly accelerates fibrosarcoma (RAF) kinases. SRF was already approved for advanced renal cell carcinoma (RCC), hepatocellular carcinoma (HCC), and advanced thyroid carcinoma [9]. SRF possesses the ability to inhibit Raf kinases within the mitogen-activated protein kinase (MAPK) pathway, which mediates cellular growth signals and is constitutively active in most UM tumours [7]. Additionally, it acts on vascular endothelial growth factor receptor (VEGFR) and platelet-derived growth factor receptor (PDGFR), causing the inhibition of tumour angiogenesis [10]. In vitro, these phenomena occur in a dose-dependent manner, showing good anti-tumoral properties: tests on a xenograft model (with UM cell line 92.1) also demonstrated SRF capability of inhibiting tumour growth and reducing metastases (33% without treatment vs. 60% with Sorafenib) [3]. Recently, Santonocito et al. demonstrated that a new nanostructured microemulsions system carrying 0.3% Sorafenib, administered as an ophthalmic formulation, is able to deliver effective amounts of Sorafenib to the retina, reducing proinflammatory and proangiogenic mediators in reliable models of proliferative retinopathies [11].

Despite all the promising results, including phase II clinical trial with SRF monotherapy in metastatic UM [8], its clinical use is limited due to its severe side effects (diarrhoea, hand-foot skin reaction, alopecia, anorexia, weight loss, and abdominal pain [10]) consequent to oral administration [12].

Furthermore, the potential application of SRF in the treatment of UM needs to face the intrinsic difficulty of the target site, which is not easy to achieve in the case of poorly water-soluble drugs [13]. In fact, reaching the eye, especially the inner part, is a challenge both through topical and systemic administration. Topical administration is limited by eye physiological barriers, such as the cornea, which limit the access of xenobiotics [14], but also by some protection mechanisms, like blinking, tearing, and drainage, which promote a quick clearance of the drug [15]. On the other hand, it is equally difficult to make a drug reach the eye from the inside, due to the blood-aqueous barrier, which works similarly to the blood-brain barrier, preventing drug diffusion from blood to retina, through the intervention of uveal capillary endothelia and ciliary epithelia. Because of all these mechanisms, the bioavailability of drugs in the eye is poor, with an absorbed dose less than 3–5% of that administered [15].

The drawbacks related to ocular treatments could be overcome through the encapsulation of drugs into nano-sized carriers, which provide a prolonged and controlled release, the possibility to reach the target site and a longer residence time enhancing corneal permeation [16].

In order to exploit SRF potentiality, different nanoencapsulation strategies have been investigated, including liquid crystalline, lipid, and polymeric nanoparticles [10], demonstrating the possibility to protect SRF from inactivation with a considerable increase of its water solubility. Among the different nanoparticles, lipid ones have been demonstrated to be suitable for ocular delivery since they are highly biocompatible [14] and their lipid components are able to interact with the outside lipid layer of the tear, promoting longer retention thus acting as a depot [15]. Particularly, solid lipid nanoparticles (SLN), but also nanostructured lipid carriers (NLC), represent promising strategies in ocular delivery since they are able to incorporate a great amount of drug (up to 90%), sustain a prolonged residence in the precorneal area and successfully encapsulate lipophilic molecules [13,14,15].

The aim of this work was the development of SRF-SLN for the potential treatment of UM. As a quali-quantitative preliminary study, two solid lipids—Softisan or Suppocire—were analysed at different concentrations and the obtained systems were characterised through Photon Correlation Spectroscopy (PCS) to determine their mean size (Zave), polydispersity (PDI) and zeta potential (ZP); also, stability over time was exploited by Turbiscan^®^ AG Station. In vitro SLN cytocompatibility was assessed on Statens Seruminstitut Rabbit Cornea (SIRC) cells using the Short Time Repeated Exposure (S.T.R.E.) protocol. In order to assess the suitability of the system for topical delivery of SRF to the posterior segment of the eye, mucoadhesive properties were evaluated on the selected SLN, optimised by the addition of two different positively charged coating layers: the cationic lipids didodecyldimethylammonium bromide (DDAB) or dioleoyl-trimethylammonium-propane (DOTAP) chloride. The optimised formulation was selected for the delivery of SRF, whose release profile was investigated.

## 2. Materials and Methods

### 2.1. Materials

Suppocire NB (C10–C18 Triglycerides) was obtained from Gattefossè and Softisan 100 (Hydrogenated Coco-Glycerides) was kindly provided by IOI Oleo (Hamburg, Germany GmbH). Tegin O (Gliceryl Monooleate) was purchased from ACEF (Piacenza, Italy). Didecyldimethylammonium bromide (DDAB), Tween^®^ 80 (Polysorbate 80), *N*-[1-(2,3-Dioleoyloxy)propyl]-*N*,*N*,*N*-trimethylammonium chloride (DOTAP), Potassium phosphate monobasic and Phosphate buffered saline were purchased from Sigma Aldrich Co (St. Louis, MO, USA). Sorafenib tosylate (SRF) was supplied from Hetero Labs Limited (Talengana, India). Sodium bicarbonate, all LC grade solvents used for high-performance liquid chromatography (HPLC) and Millex^®^ syringe filters (PP, PES, and PVDF pore size 0.22 µm, 33 mm) were purchased from Merck (Darmstadt, Germany). Regenerated cellulose membranes (Spectra/Por CE; Mol. Wet. Cutoff 3000) were supplied by Spectrum (Los Angeles, CA, USA).

Statens Seruminstitut Rabbit Cornea (SIRC) cells were obtained from LGC Standards S.r.l. (Milan, Italy). Basal Medium Eagle (BME), gentamicin, penicillin-streptomycin, L-glutamine (L-glu), Trypsin–EDTA, and Fetal Bovine Serum (FBS) were from Lonza (Euroclone S.p.A., Milan, Italy). Reagent for MTT assay (3-(4,5-dimethilthiazol-2-yl)-2,5-dipheniltetrazolium bromide), Mucin (mucin from porcine stomach type II), and sodium chloride were purchased from Sigma-Aldrich S.r.l. (Milan, Italy). Dimethyl sulfoxide (DMSO), calcium chloride dihydrate, and potassium chloride (of analytical grade) were purchased from VWR Chemicals (Milan, Italy). Benzalkonium chloride (BAK) 50% was obtained from Novo Nordisk Pharmatech A/S (Køge, Denmark).

### 2.2. Nanoparticles Preparation

A low-energy organic solvent-free phase inversion process (PIT method) was used for the preparation of the unloaded and drug-loaded SLN [17]. Based on previous studies, Tween 80 (6%, *w*/*v*) and Tegin O (3% *w*/*v*) were selected as surfactants in combination with different amounts (5, 7, 8 or 9%, *w*/*v*) of Softisan (SLN A5, A7, A8, A9) or Suppocire NB (SLN B5, B7, B8, B9), selected as solid lipids. SRF was added at different concentrations (0.8 or 1.0%, *w*/*v*) to the melted oily phase to prepare a drug-loaded SLN. The aqueous and the oil phases were separately heated until ~80 °C. The aqueous phase was added drop by drop to the lipid phase, at constant temperature and stirring speed (~650 rpm). The mixture was slowly cooled to room temperature under continuous stirring for 2 h. The selected formulation was modified adding to the melted oily phase two different positively charged lipids DOTAP or DDAB (0.15%, *w*/*v*), thus obtaining SLN A8-DP and A8-DB, respectively. In order to purify the colloidal suspensions from the excess of surfactants and non-encapsulated drug, SLN were centrifuged at 12,000 rpm for 1 h at 4 °C, using an ultracentrifuge (SL16R Centrifuge, Thermo Scientific, Rodano, Italy) and the pellet was redispersed in PBS.

### 2.3. Photon Correlation Spectroscopy (PCS)

The mean particles diameter (Zave), polydispersity index (PDI), and zeta potential (ZP) values of all prepared SLN were determined by Photon Correlation Spectroscopy (PCS) using a Zetasizer Nano ZS90 (Malvern Instruments Ltd., Malvern, England), as previously reported [17]. Samples (50 µL) were diluted in 1 mL of ultra-purified water before measurements. Each formulation was prepared six times, and each measure is the mean value of at least three measurements ± standard deviation (SD).

### 2.4. Sterilisation by Filtration

All samples were sterilised by filtration using three different types of hydrophilic membranes of 0.22 µm pore diameter: polypropylene (PP), polyethersulfone (PES) and polyvinylidene fluoride (PVDF) (Whatman, VWR Chemicals, Milan, Italy). SLN were filtered and, when possible, the obtained sterile formulations were analysed by PCS to verify particles diameter.

### 2.5. Osmolality and pH

Osmolality values of the prepared SLN were determined by an osmometer (Osmomat 3000, Gonotec, Berlin, Germany), previously calibrated with ultra-purified water and physiological solution. A pH meter (Mettler Toledo, Milan, Italy) was used to measure the pH values of the SLN.

### 2.6. Turbiscan^®^ AG Station

A Turbiscan^®^ Ageing Station (TAGS, Formulaction, L’Union, France) optical analyser was used to investigate the physical stability of unloaded-SLN suspensions. The equipment was composed of the ageing station, which allows the storage of samples in three thermo-regulated blocks [18]. The detection was operated using a pulsed near-infrared light source (λ = 880 nm), which sends information to two synchronous transmissions (T) and backscattering (BS) detectors: in particular, T detector receives the light crossing the sample at 180° from the incident beam, while the BS detector receives the light scattered backwards by the sample at 45° from the incident beam. This detection system produces 1625 acquisitions for each measurement, since it scans the entire height of the sample cell (65 mm longitude), acquiring T and BS each 40 µm. Using this instrument, it is possible to evaluate the occurrence of instability phenomena, such as particles migration or aggregation, in colloidal suspensions (liposomes, lipids, polymeric nanoparticles [18,19,20]. In this experiment, the cylindrical glass cell contained 20 mL of each unloaded formulation, and the storage temperatures of the three blocks were 25, 40, and 60 °C. The stability of the samples was measured through the analysis of the variation of transmission (ΔT).

### 2.7. Cell Viability Studies

Cytocompatibility of SLN was evaluated in Statens Seruminstitut Rabbit Cornea (SIRC) cells. SIRC cells were grown in a humidified 5% CO_2_ atmosphere at 37 °C in a complete culture medium (CCM), made of BME containing 10% FBS, 100 U/mL of penicillin-streptomycin, 10 mg/mL gentamicin, and 2 mM L-glutamine. Each well of a 96-well tissue culture plate was seeded with 40,000 cells in 100 µL of CCM. Cells were allowed to grow at 37 °C, 5% CO_2_ until subconfluence (70–90%), and then repeatedly exposed (6×) for 10 min to 100 µL of 5 mg/mL test item solutions prepared using sterile culture medium consisting of FBS-free BME. In detail, test items SLN A8, SLN B8, and SLN B9 were prepared by ultracentrifugation and resuspended in a neutral isotonic PBS (300 mOsm/kg, pH 7.2). A concentrated suspension of each nanostructured system was obtained and diluted in FBS-free BME to be successively tested on cells at a final concentration of 5 mg/mL.

Cells were also exposed to the negative control (sterile culture medium consisting of FBS-free BME, CTRL−) and positive control (0.01% BAK, CTRL+) for cytotoxicity evaluation. All samples were tested in triplicates and on two different experiment days. Treatments were removed after 10 min of exposure and all cells were re-fed with CCM. Before re-feeding, only wells included in the “wash” protocol were washed once with BME (free of FBS, antibiotics, and L-glu). The same procedure was repeated six times at intervals of 1.5 h [Short Time Repeated Exposure (S.T.R.E.)]. At the end of the repeated exposures, cells were incubated in standard conditions, and 24 h later, the medium was removed and replaced with 100 µL of MTT solution (0.2 mg MTT/mL of CCM). Following a 30 min incubation, MTT formazan was extracted with 100 µL of 100% DMSO. The optical density of the samples obtained (O.D.) was read at 570 nm in a microplate spectrophotometer (SPECTRAFluor Plus, Tecan, Männedorf, Switzerland). Cell viability was calculated as a percentage of the negative control.

### 2.8. Encapsulation Efficiency and In Vitro Drug Release

The amount of SRF encapsulated in the lipid matrix of SLN A8 was determined after centrifugation of the sample. The pellet was diluted in tetrahydrofuran (THF) and vortexed (Heidolph Reax 2000, VWR, Milan, Italy). The amount of SRF was directly determined by using HPLC (see Section 2.9) without interference from the other formulation components. The encapsulation efficiency (EE%) was calculated from the ratio between the amount entrapped inside the nanoparticles and the total amount of drug used for their preparation (Equation (1)).

EE% = (amount of entrapped drug/total amount of drug used) × 100(1)

Franz-type diffusion cells were used to analyse SRF in vitro release from SLN. Firstly, the moistening of the 0.75 cm^2^ regenerated cellulose membranes (Spectra/Por CE; Mol. Weight Cut-off 3.5 kDa) was operated through the immersion in physiological solution for 1 h at room temperature. Then, the receptor compartment of cells was filled with 4.5 mL of a mixture of the physiological solution and ethanol (50:50 *v*/*v*), thermostated at 35 ± 2 °C, and constantly stirred at 600 rpm. Despite the lack of bio relevance, 50% ethanol was mandatory to achieve SRF sink conditions in release studies, enabling its solubility, detection, and quantification. In the donor compartment, 500 µL of SRF-SLN was applied. Withdrawn extracted by receptor compartments (200 µL) were performed at scheduled time intervals (0, 1, 2, 3, 4, 5, 6, 24, 48, 72 h) and replaced with an equal volume of fresh receiving fluid equilibrated to 35 °C. This procedure was carried out at least three times for each sample. Finally, SRF contents were measured using HPLC (as described in Section 2.9).

### 2.9. High-Performance Liquid Chromatography (HPLC) Analyses

An Agilent model 1100 liquid chromatograph (Agilent, Santa Clara, CA, USA), equipped with an autosampler Agilent model 1100 and Chemstation Agilent software for data elaboration, and a reversed-phase C18 column (Luna 100, 5 µm, 150 × 4.6 mm Phenomenex, Santa Clara, CA, USA) was used to perform high-performance liquid chromatography-UV (HPLC-UV) analysis, to measure SRF contents. As a mobile phase, a mixture of 20 mM of potassium dihydrogen-phosphate aqueous solution and acetonitrile (35:65 *v*/*v*) was used, and the column flow rate was set at 1 mL/min. The detection of the effluent was conducted at λ = 260 nm, showing a retention time of 8 min. This method was verified according to International Conference on Harmonisation (ICH) guidelines (ICH Q2 (R1) Validation of analytical procedures: text and methodology). A calibration curve was produced analysing the absorption of known concentration of SRF in THF, and the obtained linear regression value was: R^2^ = 0.99987. Known amounts of SRF were spiked on SLN formulation and dissolved in THF, the absorption was determined for all solutions. No interference of the other formulation components was observed.

### 2.10. Stability and Interaction of Nanoparticles in the Presence of Ocular Mucus Component

#### 2.10.1. Physico-Chemical Evaluation

Optimisation of SLN A8 in terms of mucoadhesive properties was operated adding 0.15% *w*/*v* of DDAB (A8-DB) or DOTAP (A8-DP) to the lipid phase during nanoparticles preparation. The obtained formulations were incubated with mucin dispersion (1:1 *v*/*v*) in simulated tear fluid (STF: NaCl 0.68 g, NaHCO_3_ 0.22 g, CaCl_2_·2H_2_O 0.008 g, KCl 0.14 g, and distilled deionised water to 100 mL) at 35 °C, in order to analyse the stability and interaction of nanoparticles with mucin. Mean size, PDI, and zeta potential (ZP) of the nanoparticles/mucin dispersions were measured by Zetasizer NanoZS90 at scheduled time intervals (0, 1, and 24 h).

#### 2.10.2. Mucoadhesive Strength

Positively charged nanoparticles (A8-DB and A8-DP) were evaluated regarding their mucoadhesive strength based on the interaction with the negatively charged mucin. Briefly, equal volumes of mucin (0.1 *w*/*v* in STF) and nanoparticles were stirred for 15 min at room temperature and incubated for 1 and 24 h at 35 °C, and then centrifuged at 13,000 rpm (ThermoScientific™ SL16R, ThermoFisher Scientific, Waltham, MA, USA), for 1 h at 6 °C. A UV-VIS spectrophotometer (UH5300 UV-Visible Double-Beam Spectrophotometer, Hitachi Europe, Milan, Italy) was used to quantify the amount of free mucin in the supernatant at 228 nm. The calibration curve for the quantitative evaluation of mucin was linear in the following range: 1–0.06 mg/mL (R^2^ = 0.9891). The mucin-binding efficiency (%), expressing the mucoadhesive strength of the nanoparticles, was calculated according to Equation (2):

(2) $$\begin{array}{ccc} \mathrm{Mucin}\;\mathrm{binding} & \mathrm{efficiency}\;\% & =\frac{\mathrm{total}\;\mathrm{amount}\;\mathrm{of}\;\mathrm{mucin}-\mathrm{free}\;\mathrm{amount}\;\mathrm{of}\;\mathrm{mucin}}{\mathrm{total}\;\mathrm{amount}\;\mathrm{of}\;\mathrm{mucin}}\times 100 \end{array}$$

### 2.11. Statistical Analysis

All data from PCS are reported as mean values ± SD. Differences, analysed by two-sample hypothesis testing (*t*-test), using Origin Software (version 8.5.1), were considered statistically significant for *p* < 0.05. Cell viability data were analysed in Prism 6 (GraphPad Inc., La Jolla, CA, USA) by one-sample *t*-test (treatment vs. cut-off) assuming a cut-off value for cytotoxicity of 50% as per ECVAM protocol DB-ALM n° 17: MTT Assay (EURL ECVAM Database on Alternative Methods to Animal Experimentation. Available online: http://cidportal.jrc.ec.europa.eu/ftp/jrc-opendata/EURL-ECVAM/datasets/DBALM/LATEST/online/DBALM_docs/17_P_MTT%20Assay.pdf, accessed on 5 October 2021). For the statistical analysis of nanoparticles during stability study in mucin dispersion (STF), two-way ANOVA was performed. Multiple comparisons were performed according to Sidak’s multiple comparisons test. Analyses were performed using Prism 8 (GraphPad Software, Inc., La Jolla, CA, USA, version 8.0.2, last accession date 19 October 2021), applying *p* < 0.05 as the minimum level of significance.

## 3. Results and Discussion

### 3.1. Physicochemical Characterisation

**Figure 1:**
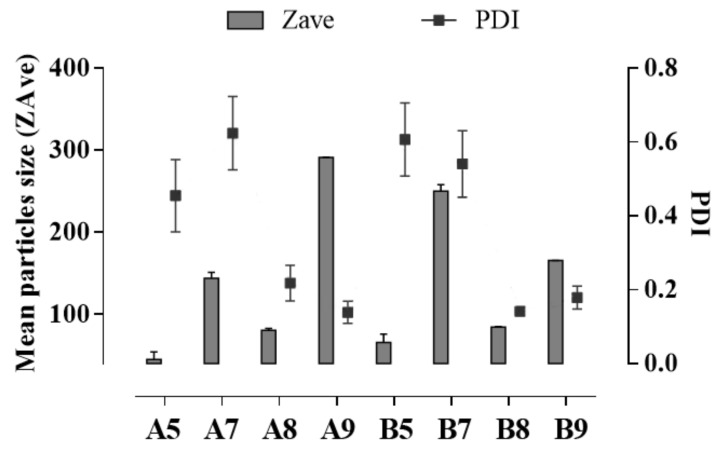
Mean particle size (Z-Ave, (nm)) and polydispersity index (PDI, (a.u.)) of SLN prepared with different amounts of Softisan (A5, A7, A8, and A9) or Suppocire (B5, B7, B8, and B9). Each measurement represents the mean value ± standard deviation (SD), *n* = 6.

As reported in the literature, nanoparticle size strongly affects drug distribution and residence time in the eye’s structure, with nanoparticles smaller than 200 nm usually showing a burst release followed by a gradual release profile in vitro and a longer half-life, compared to smaller nanoparticles characterised by a longer half-life and a sustained drug release [21,22]. In order to obtain homogeneous small-sized SLN for potential ophthalmic application, a preliminary quali-quantitative screening was developed on two different solid lipids, Softisan (A) and Suppocire (B), at different concentrations (5, 7, 8, 9 % *w*/*v*). The eco-friendly PIT method, reported in the literature as an easy method with a low impact on the environment [17], was used as a lab-scale preparation procedure. The obtained results showed that the amount of the selected lipids strongly affected nanoparticles size. Notably, none of the lipids used at 5 or 7% *w*/*v* produced well-structured SLN, since the lowest concentration (5% *w*/*v*) produced particles with a high degree of heterogeneity in size (PDI >> 0.2), probably due to an excess of surfactants, compared to the amount of the solid lipid (Figure 1). The use of 7% *w*/*v* of lipid, in both cases, led to the formation of heterogeneous particles, characterised by the presence of different peaks of size distribution, as revealed by the multiple peaks of intensity (Supplementary Figure S1).

Conversely, using higher amounts of Suppocire in SLN B8 and B9 (8 or 9% *w*/*v*) induced a better organisation of the raw materials at the interface, with the formation of smaller and more homogeneous particles, with a mean diameter below 200 nm (Figure 1). A different behaviour was observed when using 9% *w*/*v* Softisan (SLN A9), whose very bulky chemical structure led to the formation of larger nanoparticles with sizes greater than 300 nm, which is not suitable for the ophthalmic application [21,22,23]. Contrarily, SLN A8 showed homogeneous particles (PDI < 0.3) in the nanometer range (<100 nm), therefore appropriate for the topical treatment of UM. Therefore, according to the acquired data, SLN A5, A7, A9, B5, and B7 were excluded from further analysis while SLN A8, B8, and B9 were deeper characterised.

The obtained SLN showed pH values of around 6.5 ± 0.2 with an osmolality of 298 ± 0.10 mOsm/kg, without significant differences due to the type and amount of the used lipid. Since the ocular tissue can tolerate a pH value ranging from 4 to 9, and the osmolality of human tears is in the range of 280–300 mOsm/kg (although an osmolality between 200 and 450 mOsm/kg can be tolerated); therefore, the produced SLN were compatible with the tears film.

Since sterilisation is a mandatory prerequisite for all ophthalmic formulations, a preliminary study was performed by filtration [24]. In order to select the most suitable material for the lipid nanocarriers used in this study, the sterilisation was carried out using syringe filters with three different types of membranes: polypropylene (PP), polyethylene sulfone (PES), and polyvinylidene fluoride (PVDF), with average pore diameter equal to 0.22 µm. The experimental results showed that PP and PES filters are not suitable for the filtration of the SLN, as they retain the nanoparticles (data not shown) without the possibility to obtain a filtrated suspension. The use of PVDF filters, on the other hand, allowed the filtration of all SLN, regardless of the quali-quantitative composition of the lipid matrix. From the results of PCS analysis before and after filtration, it is possible to state that all systems maintain their initial characteristics unaltered (Table 1). Therefore, the produced SLN can be easily sterilised using PVDF membrane filters, characterised by great mechanical strength and dimensional stability (no deformation under weight), with better chemical stability, almost 10 times higher compared to PP and PES filters [25,26,27].

**Figure 2:**
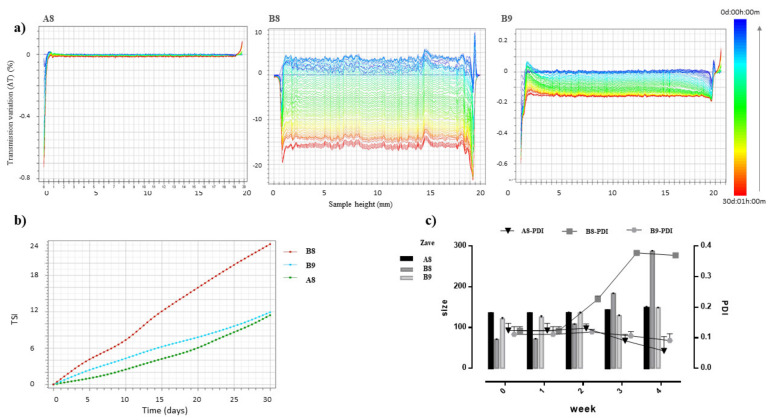
(**a**) Transmission profiles (ΔT) of SLN A8, B8 and B9 stored in Turbiscan^®^ for 30 days at 25.0 ± 1.0 °C. Data are reported as a function of time in the range 0–30 days of sample height (0 to 20 mm); (**b**) Destabilisation kinetics in terms of evolution of Turbiscan^®^ Stability Index (TSI) of samples stored in the instrument at 25.0 ± 1.0 °C for 30 days; (**c**) Mean particle size (Z-Ave, [nm]) and polydispersity index (PDI, [a.u.]) of SLN A8, B8 and B9 stored in Turbiscan^®^ and measured by PCS after 1, 2, 3 and 4 weeks. Each measurement represents the mean value ± standard deviation (SD), *n* = 6.

The physical stability of SLN A8, B8, and B9, a mandatory requirement for their potential industrial application [28], was investigated by Turbiscan^®^ AGS storing samples at room temperature (25 ± 2 °C) for 30 days (Figure 2). As shown in the graph of transmission variations (ΔT) reported in Figure 2a, SLN B8 showed important instability phenomena related to particles aggregation, highlighted by ΔT variation in the middle of the graph greater than 20%. Conversely, SLN A8 and SLN B9 showed a potential long-term physical stability, as confirmed by the absence of transmission variation in sample A8, or by the presence of insignificant transmission variation (ΔT < 20%) in SLN B9. TSI values confirmed the stability decreased in the following scale: A8 ≥ B9 >> B8 (Figure 2b).

It is worth noting that the obtained stability results were in perfect agreement with PCS measurements of samples stored in Turbiscan^®^ and analysed at different time intervals (1, 2, 3, or 4 weeks). The obtained data confirmed that SLN B8 underwent significant instability phenomena (*p* < 0.05) due to particle aggregation, whose size and PDI values increased already after 2 weeks of storage (Figure 2c, Supplementary Table S1). PCS analysis of stored samples also confirmed that SLN A8 represents the ideal formulation, among those prepared, since after 4 weeks of storage, at the 0.05 level of significance, the difference between population means was not significantly different for mean size and PDI values (Supplementary Table S1). The different behaviours of Suppocire and Softisan could be attributed to their different chemical structure and properties. Suppocire NB is a mixture of mono-, di-, and triglyceride esters of fatty acids (C10 to C18), with an intermediate melting point (35–39 °C). It rapidly recrystallises in the cooling phase of SLN production, thus determining the formation of smaller particles with harder solid core compared to the hydrogenated coco-glycerides (Softisan 100), whose lower melting range (33.5–35.5 °C) is probably responsible for the greater values of mean size at the highest concentration.

### 3.2. In Vitro Characterisation

**Figure 3:**
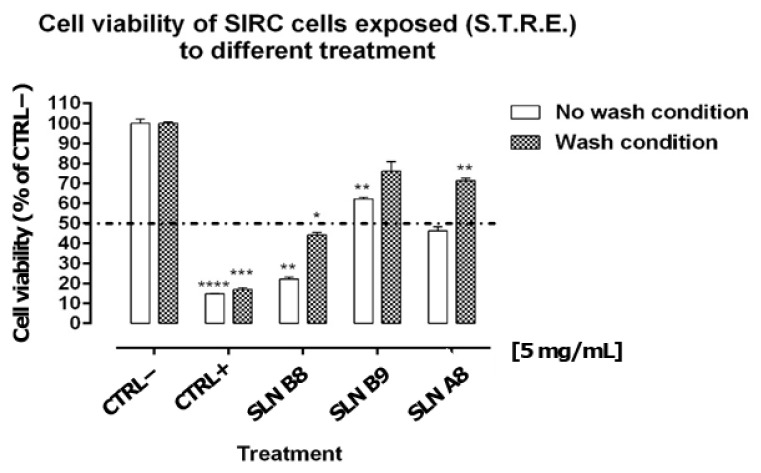
Cell viability of SIRC cells repeatedly exposed (6×) for 10 min with or without wash and with a step of 1.5 h between the repeated exposures (Short Time Repeated Exposure (S.T.R.E.)) to control formulations or delivery systems SLN B8, SLN B9, and SLN A8 diluted at 5 mg/mL. Dotted line is placed at 50% representing the cut-off value to determine cytotoxicity potential according to ECVAM protocol DB-ALM n° 17. Data represent the mean ± s.e.m. of three replicates for each condition. * *p* ≤ 0.05; ** *p* ≤ 0.01; *** *p* ≤ 0.001; **** *p* ≤ 0.0001. One sample *t*-test vs. cut-off 50%.

In order to evaluate the potential ophthalmic application of the developed formulations, the cytocompatibility profile of SLN A8, B8 and B9 was evaluated on the SIRC cell line using the method described above (see Section 2.7). Experimental results showed that BAK 0.01%, used as a positive control (CTRL+), induced significant cell mortality by 80–90%, causing the permanent loss of cell viability regardless of the “wash” or “no wash” condition (Figure 3). BAK was selected as CTRL+, considering that it is the major preservative component currently used in eye drops at concentrations even higher than 0.01% (i.e., 0.02%) [29,30]. SLN B8 produced statistically significant cytotoxic effects both in “wash” and “no wash” conditions, inducing cell mortality by 56 ± 1.09% and 78 ± 0.98%, respectively (Figure 3). Conversely, SLN A8 and SLN B9 were found to cause no significant effect in the “no wash” condition (Figure 3). Noteworthy, SLN B9, with particles of about 150 nm, showed a better cytocompatibility profile compared to smaller nanoparticles, as reported in Figure 3.

Consistently, the same systems (i.e., SLN A8 and SLN B9) were found to be endowed with particularly favourable profiles when tested in the “wash” protocol (Figure 3). Indeed, SLN A8 prepared using Softisan showed a more favourable profile compared to Suppocire NB, probably due to the lowest melting temperature of Softisan, which could provide a softer and more flexible structure, that is more favourable in cell interactions [27]. It is worth noting that the “wash” protocol should be considered the most predictive condition of potential corneal cytotoxic effects arising from administration to the surface of the eye. Indeed, concentrations of formulations/drug delivery systems on the corneal surface are strongly affected by several factors, including dilution, blinking, and drainage, ultimately affecting their overall pre-corneal retention time [31]. More importantly, SLN A8 and B9 showed a promising cytocompatibility profile even when tested with the “no wash” protocol, which is a very extreme testing condition that may prove to be inappropriate for extrapolations to the ocular environment posing issues of potential false positives for cytotoxic effects.

Indeed, standard cytotoxicity protocols, DB-ALM Protocol n° 17, prescribe 24 h of cell exposure to test items [32] and this exposure time is rather extreme if compared to eye drops residence time on the human ocular surface of only a few minutes [31]. Certainly, this approach is most likely overestimating true cytotoxic effects. Therefore, we developed the Short Time Repeated Exposure (S.T.R.E.) test using SIRC cells as an alternative method for assessing eye irritation merging notion from both the DB-ALM Protocol n° 17 and the short-time exposure test by Takahashi et al. to better mimic the real situation after administration of drugs to the ocular surface [33]. Thus, the protocol entails exposing the target cells to test items for 5 min for a total of six repetitions, summing up to 30 min of total exposure in a 12 h interval. Hence, we have simulated both realistic corneal residence times and repeated administration courses typically associated with ophthalmic eye drops treatments.

In order to improve the retention time after topical administration, SLN A8 was optimized with the addition of a positively charged lipid, and the effect of two monovalent cationic lipids with two lipophilic chains was investigated by mucoadhesive studies. In particular, DOTAP was selected as an FDA-approved material and, in order to reduce the cost of the final product, its effect was compared to that of DDAB, selected for its lowest cost and ability to provide a coating layer on lipid nanoparticles [34,35].

The addition of DDAB (A8-DB) or DOTAP (A8-DP) as coating layer did not significantly modify mean particles size and PDI values of the SLN A8 while, as expected, ZP values turned from negative to highly positive values (+20 mV). The stability of colloidal particles in biological fluids containing relevant levels of proteins is a crucial issue. Currently, it is broadly accepted that the size of the particles plays an important role in their ability to interact with cells and in their transport through the mucus layer, such as that of the ocular mucosa [36]. Surprisingly, despite the importance of the size, there are very few articles on the stability of colloidal particles in biological fluids. For example, Tobìo et al. showed that poly (lactic acid) nanoparticles aggregated significantly upon contact with simulated gastric fluids [37]. Similarly, it was observed that poly-ε-caprolactone nanocapsules suffered an immediate aggregation process upon their incubation with lysomes [38].

In order to investigate nanoparticles behaviour after ophthalmic administration, experiments were performed in presence of mucin, which is one of the main components of the ocular mucus layer, dispersed in STF, thus investigating the effect of proteins and ions on the stability and the mucoadhesive strength of nanoparticles intended for ocular delivery. Experiments were performed comparing the effects of the two different coating lipids (DDAB and DOTAP) to the uncoated negatively charged SLN A8.

**Figure 4:**
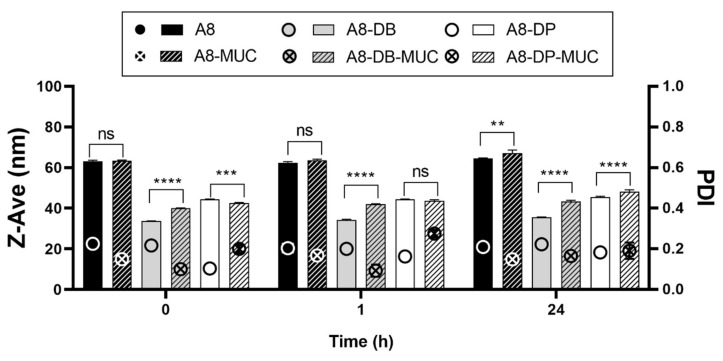
Nanoparticles mean size (Z-Ave, bars) and PDI (symbols) before (A8, A8-DB, A8-DP) and after (A8-MUC; A8-DB-MUC; A8-DP-MUC) 1 and 24 h of incubation with mucin at 35 °C. Significance was set as *p* > 0.05; ** *p* ≤ 0.01; *** *p* ≤ 0.001; **** *p* ≤ 0.0001; ns—not significant.

As reported in Figure 4, statistically significant variations in particle mean size can be observed for nanoparticles/mucin dispersions over time compared to the A8, A8-DB, and A8-DP controls, with the following trend A8-DB > A8-DP > A8. This result suggested that the main interaction was established between A8-DB and mucin at all time points investigated, followed by A8-DP and as expected, the negatively charged SLN A8. Looking carefully at these results, we can observe that the increase in particle mean size for nanoparticles incubated in mucin dispersion is associated with PDI values ≤ 0.275 indicating the absence of aggregation and/or precipitation phenomena, thus revealing good stability in a simulated biological fluid (STF with mucin).

**Figure 5:**
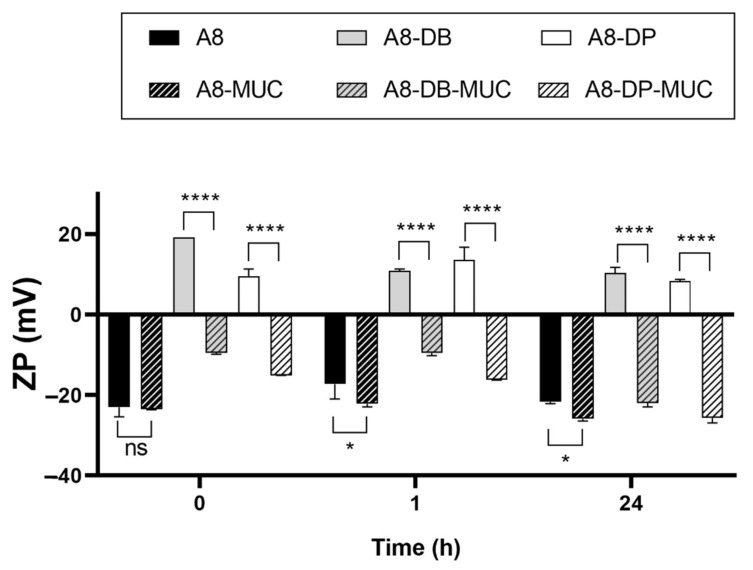
Nanoparticles zeta potential (ZP) before (A8, A8-DB, A8-DP) and after (A8-MUC; A8-DB-MUC; A8-DP-MUC) 1 and 24 h of incubation with mucin at 35 °C. Significance was set as *p* > 0.05; * *p* ≤ 0.05; **** *p* ≤ 0.0001; ns—not significant.

ZP measurement is a common method to investigate the mucoadhesive properties of several biopolymers [39] and can be also used to evaluate the biophysical interactions of lipid nanoparticles with mucin [40], which has a negative charge. For this reason, the superficial charge of nanoparticles is an important parameter since repulsion between nanoparticles and mucin occurs with negatively charged systems while there will be an attraction to the negatively charged mucin and the positive particles [40]. Mucin has a negative charge (approximately −7 mV), thus the positive surface charges of A8-DB and A8-DP formulations are expected to strongly interact [41]. Accordingly, positive ZP values of A8-DB and A8-DP were inverted to negative values after incubation with mucin (Figure 5), suggesting that strong interactions between mucin and nanoparticles occurred, thus confirming our previous results (Figure 4).

The electrostatic interaction is the most expectable mucoadhesive mechanism. The decrease in ZP for both nanoparticles before and after incubation with mucin supports this observation. The occurrence of ionic interactions caused a decrease in the ZP value because mucin interacted with the positively charged surface layer of nanoparticles, neutralizing the positive charges [42]. Contrarily, the ZP of SLN A8 remained almost unchanged in the presence of mucin, or decreased furtherly and reached a relatively large negative value as a result of weaker interactions [19] and the absence of bridging effect [16], since no or low change (** *p* ≤ 0.01) in particle size was observed (Figure 5). Based on these findings, the mucoadhesive strength of positively charged nanoparticles was furtherly investigated. Both samples (A8-DB and A8-DP) exhibited relevant mucin-binding efficiency concomitant with the conversion in the ZP values, as presented in Figure 5. The sample A8-DB possessed the maximum mucin-binding efficiency (%) (99.2 ± 1.08%), compared to A8-DP (61.5 ± 2.34%). These results highlighted a different ability of the two cationic lipids to interact with mucin, with DDAB having greater mucoadhesive properties compared to DOTAP. This effect could be related to their different chemical structure, also affecting their physical state at room temperature (DDAB exists as gel while DOTAP as liquid crystalline): both aliphatic chains of DDAB are saturated, unlike DOTAP, whose chains present a double bond in the C9 position [43]. The mucoadhesive properties are correlated with the ionic interaction between the monovalent cationic lipid and the negatively charged sialic acid groups of eye mucin, with subsequent formation of non-covalent bonds [42]. Such interaction promotes the ocular residence time and cellular uptake of nanoparticles, which is essential for effective mucosal delivery of therapeutics, enhancing drug bioavailability, through the increased rate of absorption, and drug targeting.

Based on the obtained results, considering the overall behaviour, SLN A8-DB was selected for the encapsulation of SRF, a drug approved for the treatment of kidney cancer and advanced liver cancer [44,45,46,47,48,49,50] that has been recently investigated for the potential treatment of uveal melanoma and retinal neovascular diseases [2,3,7,8,11,51,52,53].

As shown in Table 2, the addition of 0.8 or 1.0 % *w*/*v* of SRF to A8 did not modify the main properties of the SLN in terms of mean particle size and homogeneity.

Both SRF-loaded nanosuspensions showed pH and osmolality values compatible with the eye and high value of EE%, corresponding to 74.5 and 75% for A8-SRF 1% and A8-SRF 0.8%, respectively. This result is consistent with literature findings investigating SRF encapsulation into lipid nanoparticles for the treatment of liver cancer, with lower EE% values found in our study probably related to the smallest size of SLN A8 nanoparticles [13,54,55,56].

**Figure 6:**
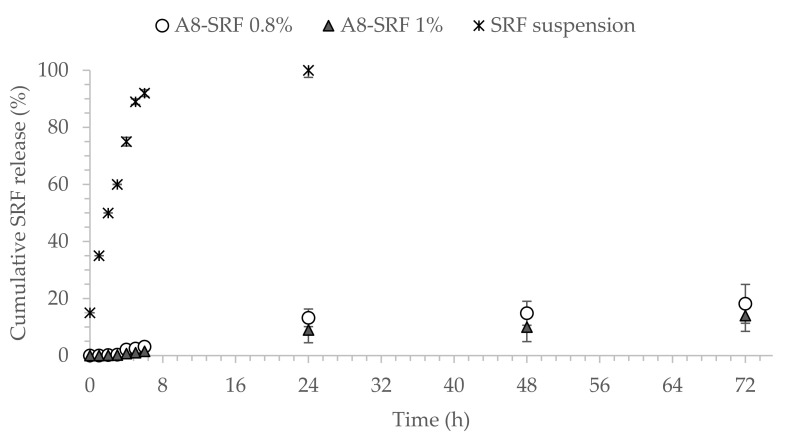
In vitro release profiles of Sorafenib (SRF) from SLN A8 loaded with 0.8% or 1.0% *w*/*v*, compared to free drug suspension 1%, *w*/*v* (mean ± SD, *n* = 3).

The cumulative release rate was calculated according to the released SRF amount compared to the total SRF amount. Figure 6 shows the release profile of SRF from SLN A8 loaded with 0.8 or 1.0 % *w*/*v* and SRF-suspension in vitro. Interestingly, SLN A8 at both concentrations provided a controlled prolonged release of the encapsulated drug, with less than 25% of SRF released after 72 h (Figure 6). The slow release may be attributed to the good encapsulation and surface coating by the lipid layer [54,57]. These results are encouraging for the potential ophthalmic application and in accordance with previous findings not only related to lipid nanoparticles [58,59,60] but also reported for polymeric nanoparticles, which have been demonstrated to provide sustained release for up to 50 days when injected intravitreally in rabbits [61].

## 4. Conclusions

The results obtained in our study demonstrated that SLN prepared using Softisan as solid lipid represents a promising strategy for the topical delivery of Sorafenib in the potential treatment of uveal melanoma due to the presence of small-sized nanoparticles, characterised by stability over time, good cytocompatibility, and high mucoadhesive properties. The developed strategy opens the perspective of the potential ophthalmic treatment of a disease that, to date, is mainly treated with systemic therapies or specific treatments directed to the liver, with a consequently positive impact on patient compliance. However, further studies need to be developed to deepen the potentiality of SRF-loaded SLN A8-DB, with particular attention on the possibility of obtaining a final freeze-dried product to be investigated in terms of stability, in vitro release, in vitro biocompatibility on SIRC, and effectiveness on a model of uveal melanoma.

## Figures and Tables

**Table 1:** Mean particles size (Z-Ave, (nm)) and polydispersity index (PDI, (a.u.)) ± standard deviation (SD) of SLN before and after filtration by PVDF membrane filters of 0.22 µm. Data reported are the mean of six different experiments. * Significance for *p* > 0.05.

Filtration	SLN	Z-Ave ± S.D. (nm)	PDI ± S.D.
Not filtered	A8	96.63 ± 2.05	0.177 ± 0.020
	B8	70.67 ± 2.08	0.126 ± 0.003
	B9	123.5 ± 1.36	0.139 ± 0.012
Filtered (PVDF)	A8	95.53 ± 0.63	0.197 ± 0.080
	B8	72.03 ± 0.46	0.178 ± 0.008 *
	B9	117.18 ± 0.82	0.165 ± 0.020

**Table 2:** Mean particles size (Z-Ave, [nm]) and polydispersity index (PDI, (a.u.)), pH, osmolality (mOsm/Kg) and encapsulation efficiency (EE%) ± standard deviation (SD) of SLN A8 loaded with 0.8 or 1.0% *w*/*v* of Sorafenib (SRF). Each value is the average of six different replicates ± standard deviation (SD).

SLN	Z-Ave ± S.D. (nm)	PDI ± S.D.	pH ± S.D.	Osmolality (mOsm/Kg) ± S.D.	EE% ± S.D.
A8-SRF 0.8%	127.85 ± 1.50	0.215 ± 0.014	6.33 ± 0.85	308 ± 2	75.0 ± 2.1
A8-SRF 1%	150.12 ± 1.85	0.180 ± 0.013	6.25 ± 0.72	302 ± 3	74.5 ± 2.8

## Data Availability

Not applicable.

## Supplementary Materials

The following are available online at https://www.mdpi.com/article/10.3390/pharmaceutics13111956/s1, Figure S1: Intensity correlation functions of light scattered at 90° and hydrodynamic radius distribution by intensity of SLN prepared with different amount of Softisan (A5, A7, A8 and A9) or Suppocire (B5, B7, B8 and B9), Table S1: Mean particles size (Z-Ave, [nm]) and polydispersity index (PDI, [a.u.]) ± standard deviation (SD) of unloaded SLN A8, B8 and B9 stored in Turbiscan® at 25.0 ± 1.0 °C and analysed at different time intervals. Each value is the average of six different replicates ± standard deviation (SD). * Significance for *p* < 0.05, compared to the initial value.
