# Supplementary material for: Sorafenib Repurposing for Ophthalmic Delivery by Lipid Nanoparticles: A Preliminary Study
Source: Pharmaceutics. 2021 Nov 18;13(11):1956. doi: 10.3390/pharmaceutics13111956 (PMC8622456; doi:10.3390/pharmaceutics13111956)
Supplement: Supplementary file 1 [file pharmaceutics-13-01956-s001.zip › pharmaceutics-1453261 Supplementary Material updated.pdf]

pharmaceutics

MDPI

Supplementary Materials: Sorafenib Repurposing for Ophthalmic Delivery by Lipid Nanoparticles: a Preliminary Study

Angela Bonaccorso, Veronica Pepe, Cristina Zappulla, Cinzia Cimino, Angelo Pricoco, Giovanni Puglisi, Francesco Giuliano, Rosario Pignatello and Claudia Carbone

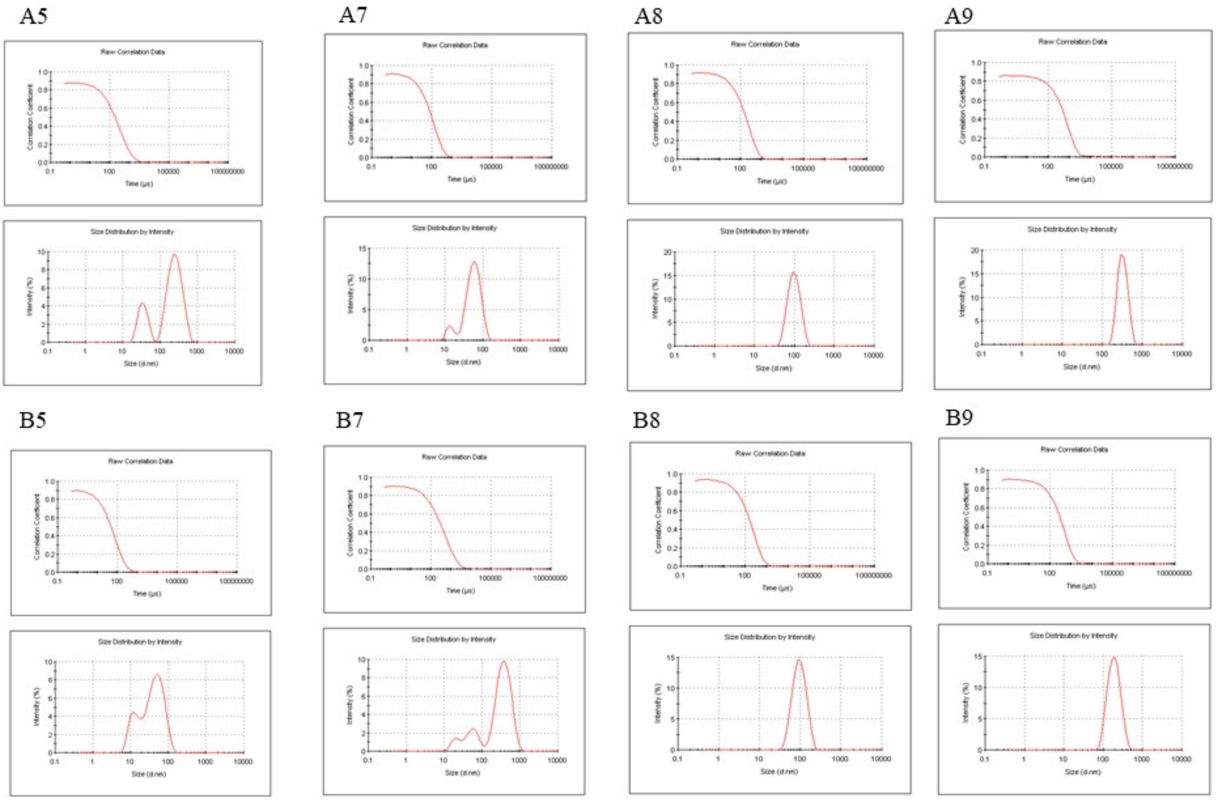

Figure S1. Intensity correlation functions of light scattered at 90° and hydrodynamic radius distribution by intensity of SLN prepared with different amount of Softisan (A5, A7, A8 and A9) or Suppocire (B5, B7, B8 and B9).

Table S1. Mean particles size (Z-Ave, [nm]) and polydispersity index (PDI, [a.u.]) ± standard deviation (SD) of unloaded SLN A8, B8 and B9 stored in Turbiscan® at 25.0 ± 1.0 °C and analysed at different time intervals. Each value is the average of six different replicates ± standard deviation (SD).

	Sample	A8	B8	B9
**Zave ± S.D. (nm)**	1 week	135.1 ± 1.15	71.60 ± 1.12	125.5 ± 3.11
	2 weeks.	135.8 ± 1.7	108.4 ± 0.4	145.6 ± 1.8
	3 weeks	138.7 ± 0.4	183.1 ± 1.4*	129.1 ± 1.4
	4 weeks	136.5 ± 2.9	287.4 ± 1.3*	156.1 ± 0.8
**PDI ± S.D.**	1 week	0.124 ± 0.023	0.123 ± 0.013	0.111 ± 0.025
	2 weeks	0.131 ± 0.014	0.226 ± 0.011	0.119 ± 0.009
	3 weeks	0.090 ± 0.021	0.378 ± 0.003*	0.106 ± 0.014
	4 weeks	0.057 ± 0.047	0.370 ± 0.004*	0.091 ± 0.022

* Significance for $p < 0.05$, compared to the initial value.
